# Supplementary material for: Variants in ASPH cause exertional heat illness and are associated with malignant hyperthermia susceptibility
Source: Nat Commun. 2022 Jun 13;13:3403. doi: 10.1038/s41467-022-31088-8 (PMC9192596; doi:10.1038/s41467-022-31088-8)
Supplement: Supplementary file 1 — Supplemental Information [file 41467_2022_31088_MOESM1_ESM.pdf]

Supplementary Table 1: Genes associated with EC-coupling

Protein	Gene Name	NCBI Gene ID
junctin	*ASPH*	444
ATPase sarcoplasmic/endoplasmic reticulum Ca2+ transporting [illegible]	*ATP2A1*	487
ATPase sarcoplasmic/endoplasmic reticulum Ca2+ transporting [illegible]	*ATP2A2*	488
ATPase plasma membrane Ca2+ transporting 3	*ATP2B3*	492
calcium voltage-gated channel subunit alpha1 S	*CACNA1S*	779
calcium voltage-gated channel auxiliary subunit alpha2delta 1	*CACNA2D1*	781
calcium voltage-gated channel auxiliary subunit beta 1	*CACNB1*	782
calcium voltage-gated channel auxiliary subunit gamma 1	*CACNG1*	786
calmodulin 1	*CALM1*	801
calcium/calmodulin dependent protein kinase II alpha	*CAMK2A*	815
calcium/calmodulin dependent protein kinase II beta	*CAMK2B*	816
calcium/calmodulin dependent protein kinase II delta	*CAMK2D*	817
calcium/calmodulin dependent protein kinase II gamma	*CAMK2G*	818
calsequestrin 1	*CASQ1*	844
calsequestrin 2	*CASQ2*	845
FKBP12	*FKBP1A*	2280
junctophilin 1	*JPH1*	56704
junctophilin 2	*JPH2*	57158
junctional sarcoplasmic reticulum protein 1	*JSRP1*	126306
ryanodine receptor 1	*RYR1*	6261
SH3 and cysteine rich domain 3	*STAC3*	246329
triadin	*TRDN*	10345

Supplementary Table 2: Identified variants in Canadian MH cohort

source	chr	start	end	ref_allele	alt_allele	var_type	typeseq	gene_symbol	ExAC_freq	sift_score	polyphen_score	CADD_phred
WGS	1	116247812	116247812	C	G	snp	intronic; splicing	CASQ2	0	NA	NA	27.6
WGS	1	160171036	160171036	C	T	snp	exonic	CASQ1	1.00E-04	0.046	0.991	24.2
WES	1	201009083	201009091	AGTAGCTCT	A	del	exonic	CACNA1S	0.0017	NA	NA	42
WGS	1	201012542	201012542	C	T	snp	exonic	CACNA1S	8.24E-06	0.012	0.118	27
WES	1	201022387	201022387	C	T	snp	exonic	CACNA1S	0.0017	0.002	0.99	34
WES	1	201046058	201046058	C	T	snp	exonic	CACNA1S	0.0088	0.002	0.859	26.8
WES	1	201046058	201046058	C	T	snp	exonic	CACNA1S	0.0088	0.002	0.859	26.8
WES	1	201058513	201058513	C	T	snp	exonic	CACNA1S	0.0074	0.096	0.938	23.2
WES	1	201058513	201058513	C	T	snp	exonic	CACNA1S	0.0074	0.096	0.938	23.2
WES	1	201058513	201058513	C	T	snp	exonic	CACNA1S	0.0074	0.096	0.938	23.2
WES	7	81601108	81601108	C	T	snp	exonic	CACNA2D1	0.0027	0.266	0.005	17.75
WGS	8	62577998	62577998	C	G	snp	exonic	ASPH	0	0.004	0.824	20.2
WES	8	62596603	62596603	A	G	snp	exonic	ASPH	9.91E-05	0.046	0.998	29.2
WES	8	62596603	62596603	A	G	snp	exonic	ASPH	9.91E-05	0.046	0.998	29.2
WES	12	110783861	110783861	G	A	snp	exonic	ATP2A2	9.88E-05	0.127	0.064	20.5
WES	12	110784210	110784210	A	G	snp	exonic; intronic	ATP2A2	0.001	0.619	0.001	NA
WES	12	110784210	110784210	A	G	snp	exonic; intronic	ATP2A2	0.001	0.619	0.001	NA
WGS	16	28906222	28906222	A	G	snp	exonic	ATP2A1	3.00E-04	0.177	0.001	17.08
WES	17	37342222	37342222	C	T	snp	exonic; intronic	CACNB1	2.57E-05	0.001	0.535	18.7
WES	19	2252525	2252525	G	A	snp	exonic	JSRP1	0.0012	0.289	0.51	22.4
WES	19	38934255	38934255	C	T	snp	exonic	RYR1	8.27E-06	0	1	27.4
WES	19	38939352	38939352	G	A	snp	exonic	RYR1	0	0	0.992	32
WGS	19	38946145	38946145	A	T	snp	exonic	RYR1	0	0	1	27.9
WGS	19	38948193	38948193	C	A	snp	exonic	RYR1	0	0.002	0.973	29.4
WES	19	38948227	38948227	C	T	snp	exonic	RYR1	2.47E-05	0	0.999	35
WES	19	38956907	38956907	G	A	snp	exonic	RYR1	4.53E-05	0.023	0.992	28.9
WES	19	38956907	38956907	G	A	snp	exonic	RYR1	4.53E-05	0.023	0.992	28.9
WES	19	38957032	38957032	G	A	snp	exonic	RYR1	0	0.051	0.736	22.7
WES	19	38958397	38958397	G	A	snp	exonic	RYR1	0.0017	0.593	0.717	18.06
WES	19	38965975	38965975	A	G	snp	exonic	RYR1	0.0051	0.34	0.202	22.5
WES	19	38968434	38968434	G	A	snp	exonic	RYR1	8.27E-06	0.27	0.998	24.4
WES	19	38976331	38976331	G	A	snp	exonic	RYR1	0.0015	0.079	0.999	25.8
WES	19	38976331	38976331	G	A	snp	exonic	RYR1	0.0015	0.079	0.999	25.8
WGS	19	38985205	38985205	G	A	snp	exonic	RYR1	0	0	0.998	30
WGS	19	38989863	38989863	G	A	snp	exonic	RYR1	0	0.001	0.994	24.9
WGS	19	38989863	38989863	G	A	snp	exonic	RYR1	0	0.001	0.994	24.9
WES	19	38989881	38989881	A	G	snp	exonic	RYR1	0.0011	0.053	0.98	18.13
WES	19	38989881	38989881	A	G	snp	exonic	RYR1	0.0011	0.053	0.98	18.13
WGS	19	38990633	38990633	G	A	snp	exonic	RYR1	2.48E-05	0	1	23.8
WES	19	38993528	38993528	G	A	snp	exonic	RYR1	8.37E-05	0.099	0.994	26.6
WGS	19	38993564	38993564	T	G	snp	exonic	RYR1	0	0.001	0.994	23.5
WGS	19	38995404	38995404	T	G	snp	exonic	RYR1	0	0	0.997	24.2
WGS	19	38995404	38995404	T	G	snp	exonic	RYR1	0	0	0.997	24.2
WES	19	38996015	38996015	C	T	snp	exonic	RYR1	1.98E-05	0.046	0.995	24.5
WGS	19	39002913	39002913	G	A	snp	exonic	RYR1	6.00E-04	0.001	0.998	23.5
WGS	19	39009877	39009877	C	T	snp	exonic	RYR1	7.48E-05	0.003	0.796	25.6
WES	19	39068824	39068824	G	A	snp	exonic	RYR1	5.32E-05	0	1	27.9
WES	20	42788571	42788571	T	C	snp	exonic	JPH2	0.0013	0.003	0.449	24.2

Supplementary Table 3. Clinical information

Cohort	Canada		UK	Canada				UK
	Proband	Probands' sister	Proband	Proband	Probands' sister	Proband's son	Proband's niece	Proband
Mutation in *ASPH*	p.Val54Ala heterozygous	p.Val54Ala heterozygous	p.Lys88Thr heterozygous	p.Asp149His heterozygous	N/A	N/A	No mutation in ASPH	p.Lys202Arg heterozygous
MH episode	-	-	-	+ (clinical grading scale score: 43)	-	-	-	-
EHS episode	-	-	1 episode: collapse & loss of consciousness during training on deployment (non-temperate region). Diagnosed with heat stroke - hospital records unavailable	-	-	-	-	At least 2 episodes of heat illness involving dizziness, visual disturbance, unsteady gait during military training runs in the UK. Responded to active cooling in the field. No reliable core temperature reading.
Muscle symptoms	Muscle cramps, twitches, spasm and stiffness, worsened with heat and exercise	Muscle cramps, twitches, spasm and stiffness, worsened with heat and exercise	none	Severe muscle cramps and twitches all over his body, extensive muscle pain affecting his functional ability	Nothing	Very mild muscle cramps	Nothing	none
Heat sensitivity	Muscle symptoms worsened with heat and exercise	Muscle symptoms worsened with heat and exercise	none	Muscle symptoms worsened with heat, excessive sweating	Nothing	Nothing	Nothing	With exercise
CK	High (500-1000 IU/L)	N/A	normal	200-300 IU/L	Normal	Normal	Normal	normal
Histopathology	Normal	Normal	normal	Normal	Normal	Normal	Normal	normal
Contracture test	MHShc by CHCT (caffeine: 0.4*, halothane:1.4**)	MHShc by CHCT (caffeine: 0.8*, halothane:1.0**)	MHN by IVCT laboratory classification (halothane 0.05, caffeine 0)	MHShc by CHCT (caffeine: 0.6*, halothane: 4.0**)	MHS by CHCT	MHSh by CHCT (caffeine: 0.1, halothane: 1.4**)	MHSh by CHCT (caffeine: 0.2, halothane: 1.8**)	MHN by IVCT laboratory classification (halothane 0.1, caffeine 0)
Heat tolerance test	Not performed	Not performed	Failed on 2 occasions	Not performed	Not performed	Not performed	Not performed	Failed on 2 occasions
Therapy	Oral dantrolene with favorable results	Oral dantrolene with favorable results	Discharged from military service	Oral dantrolene responded partially				Discharged from military service

The threshold responses for a positive diagnosis in CHCT were *caffeine>0.3 and/or **halothane>0.7.
The threshold for a positive diagnosis in IVCT were 0.2 g force produced at 2 mM caffeine and/or at 2%.

Supplementary Table 4. Numbers of myotubes analyzed in Figure 3a

Caffeine (mM)	0.3	0.7	1	3	10	30
Tg-WT	26	7	6	7	3	3
Tg-V54A	35	10	10	9	3	3
Tg-K88T	51	11	12	13	7	8

Supplementary Figure 1: Transcript isoforms of *ASPH*. Data Source: GTEx Analysis Release V8 (dbGaP Accession phs000424.v8.p2)

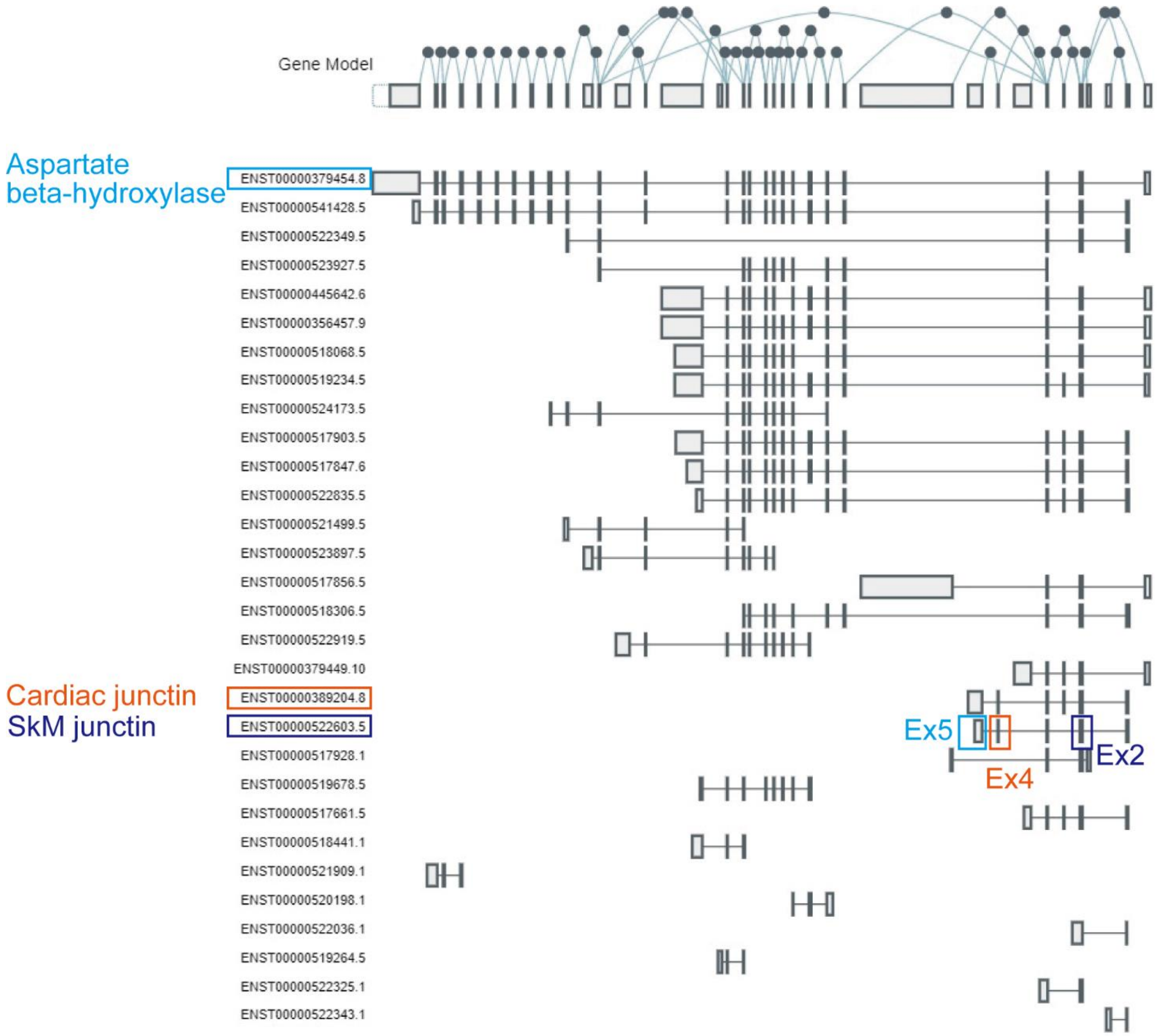

Supplementary Figure 2: The location of variants identified in Junctin protein.

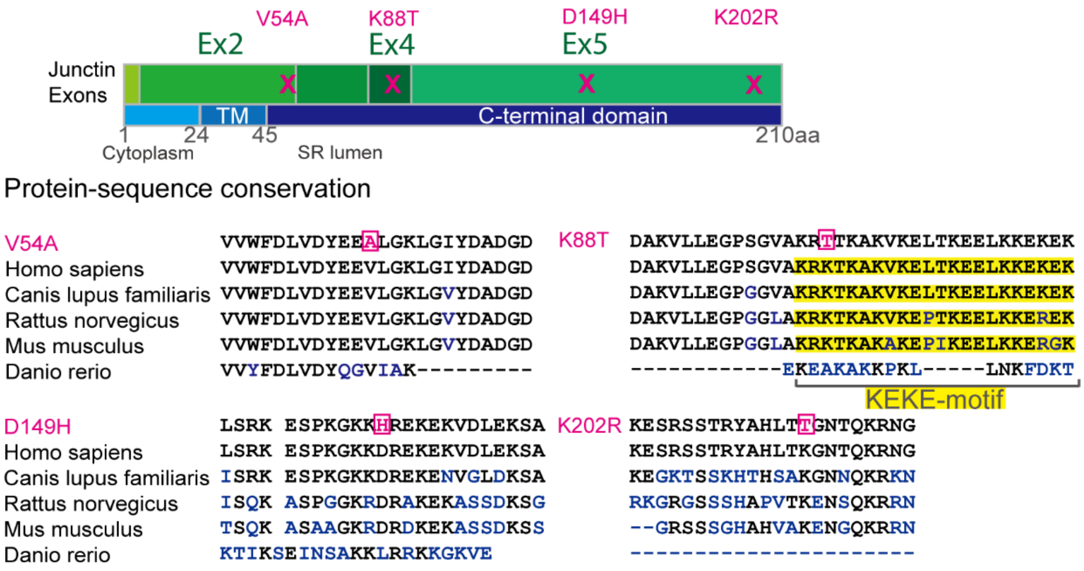

Supplementary Figure 3: mRNA levels of mCherry-junctin in Tg-fish. Three technical replicates of each sample and four independent replicates were analyzed statistically by one-way ANOVA followed by Dunnett's multiple comparisons test. Error bars represent mean ± SEM. Differences were considered to be statistically significant at $P < 0.05$ (*). Source data are provided as a Source Data file.

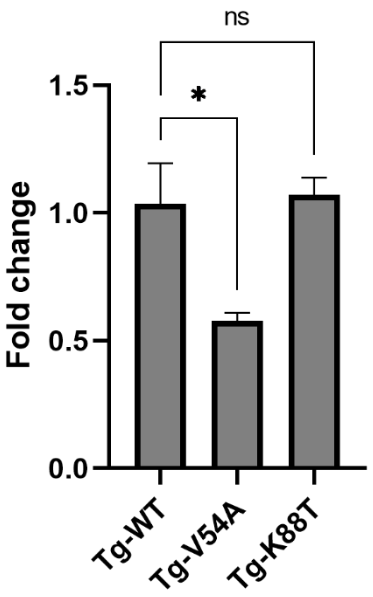

Supplementary Figure 4: Swim analysis after heat plus caffeine exposure. Tg-fish **(a)** and RNA-injected fish **(b)** were assessed at normal temperature (28.5°C) and after exposure to heat plus 1 mM caffeine (34°C & caffeine). The means of independent replicates were normalized to the means of control from the same day and experiment. Five independent replicates were analyzed statistically by one-way ANOVA followed by Dunnett's multiple comparisons test. Error bars represent mean ± SEM. Differences were considered to be statistically significant at $P < 0.05$ (*) or $P < 0.01$ (**). Source data are provided as a Source Data file.

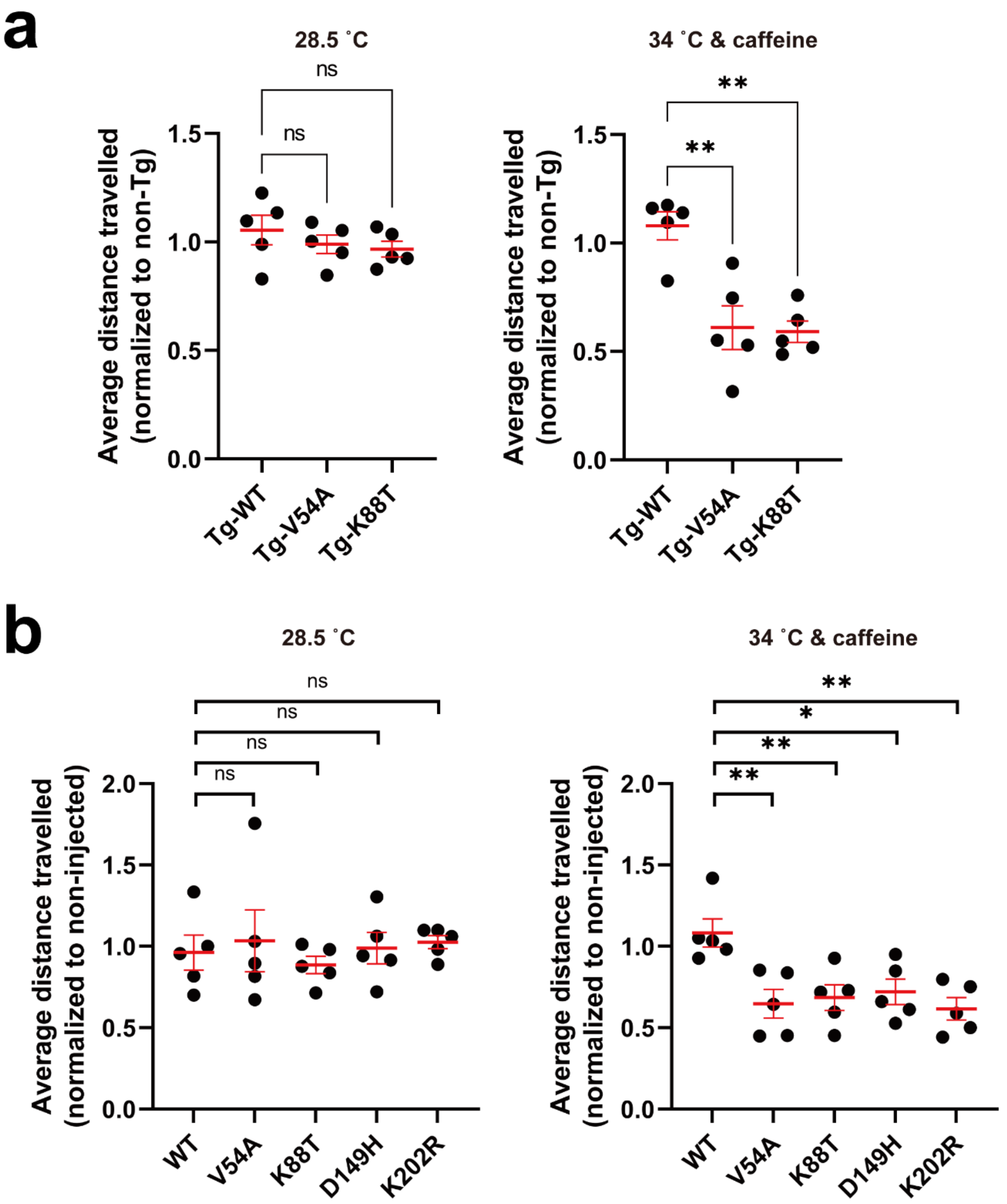

Supplementary Figure 5: Swimming performance of non-Tg fish used for normalization in Figure 3c. Scatter plots display the swimming distance of 72 fish per a group (from 3 independent replicates of 24 fish) with a normalization to the mean of the group on the same day. Source data are provided as a Source Data file.

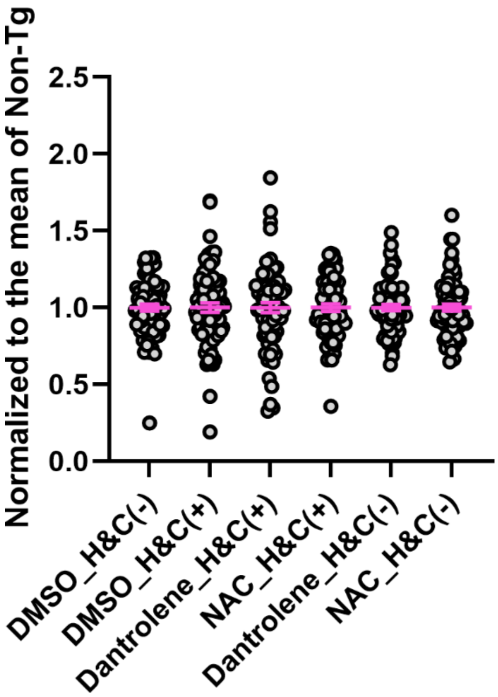

Supplementary Figure 6: Generating disease model using C2C12 cells by CRISPR/Cas9 mutagenesis. The genomic DNA sequence of established cell lines was analyzed by Sanger methods.

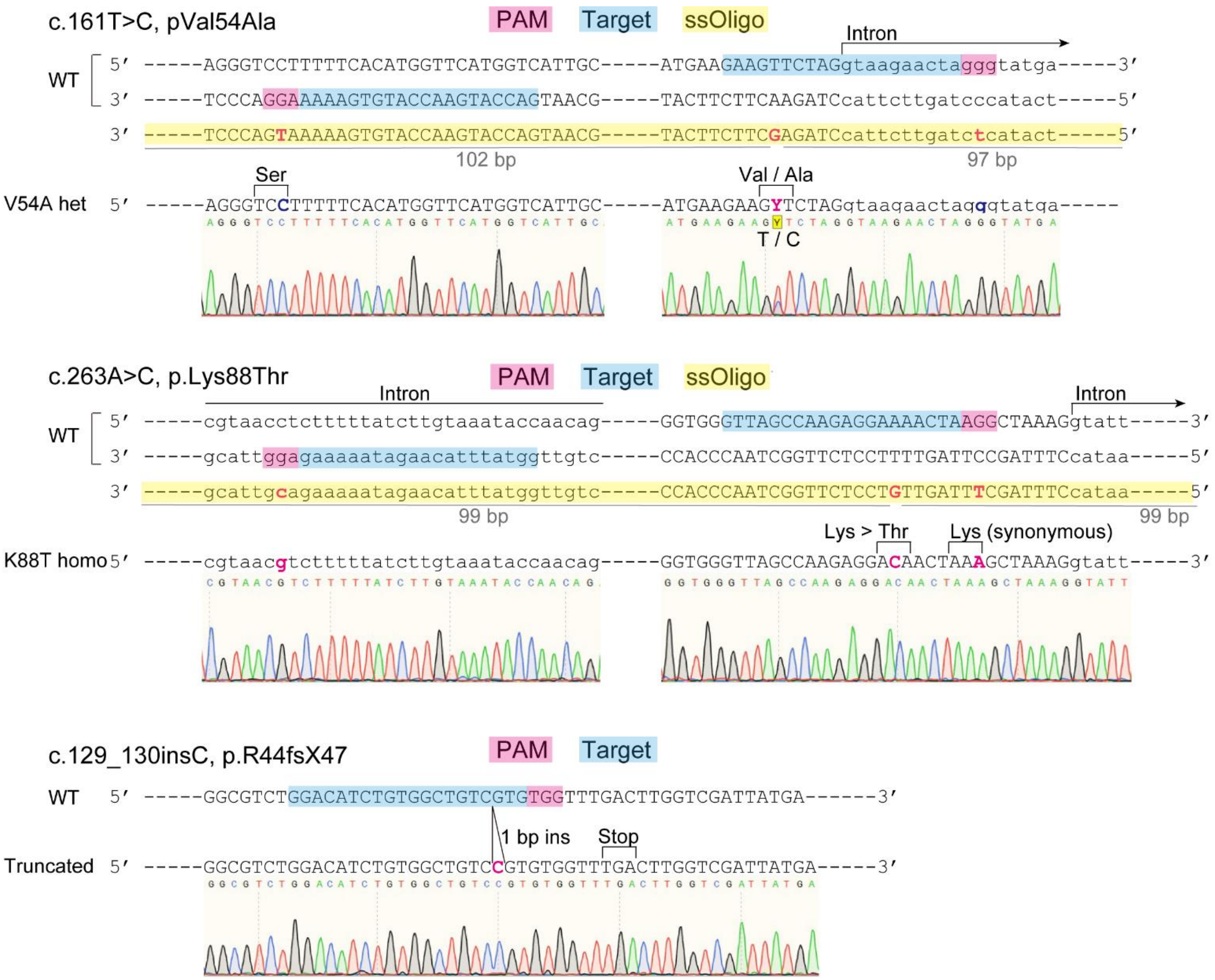

Supplementary Figure 7: Western blotting using C2C12 myotubes. a, Full images of the protein-transferred membrane. **b,** Quantifications of protein levels included three independent replicates. Junctin and Myosin were run on the same blot. Blots for Casq1 and Ryr1 were processed in parallel using the samples derived from the same experiment. For normalization of RYR1 expression levels, we used the total protein data from the blots for junctin since blots for RyR1 detected only the larger proteins and did not reflect the full total amount of proteins in the samples. Data were analyzed statistically by one-way ANOVA followed by Dunnett's multiple comparisons test. Error bars represent mean ± SEM. Differences were considered to be statistically significant at $P < 0.05$ (*), $P < 0.01$ (**), $P < 0.001$ (***) or $P < 0.0001$(****). Source data are provided as a Source Data file.

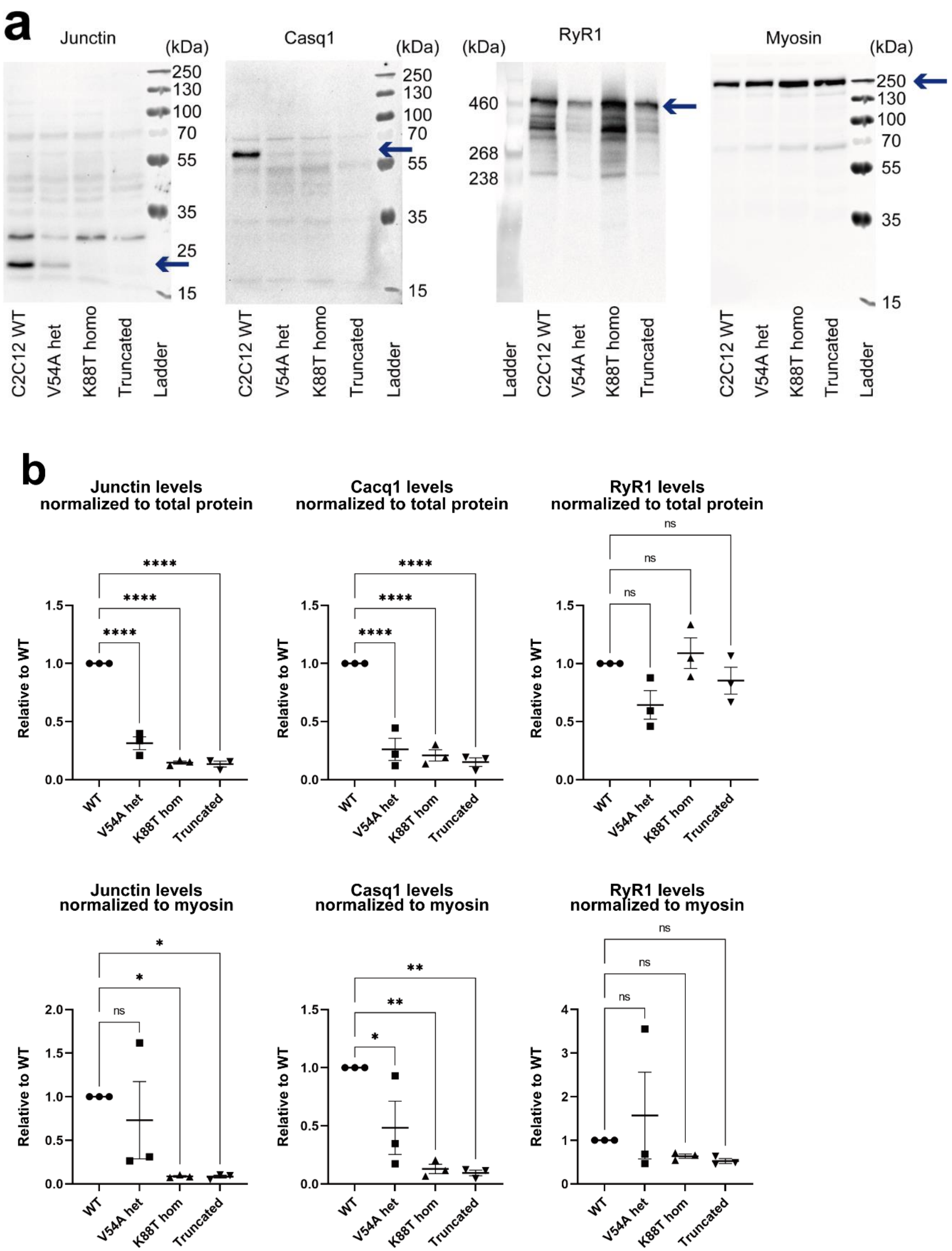

Supplementary Figure 8: DCFDA assay in C2C12. ROS in 6-day myotubes was measured at stable temperature (25°C or 37°C) with treatment (1 mM caffeine or 50 μM 4CmC) for 30 min. Statistical analysis of three independent replicates by two-way ANOVA followed by Dunnett's multiple comparisons test. All data are represented as mean ± SEM. Differences were considered to be statistically significant at P < 0.05 (*), P < 0.01 (**), P < 0.001 (***) or P < 0.0001(****). Source data are provided as a Source Data file.

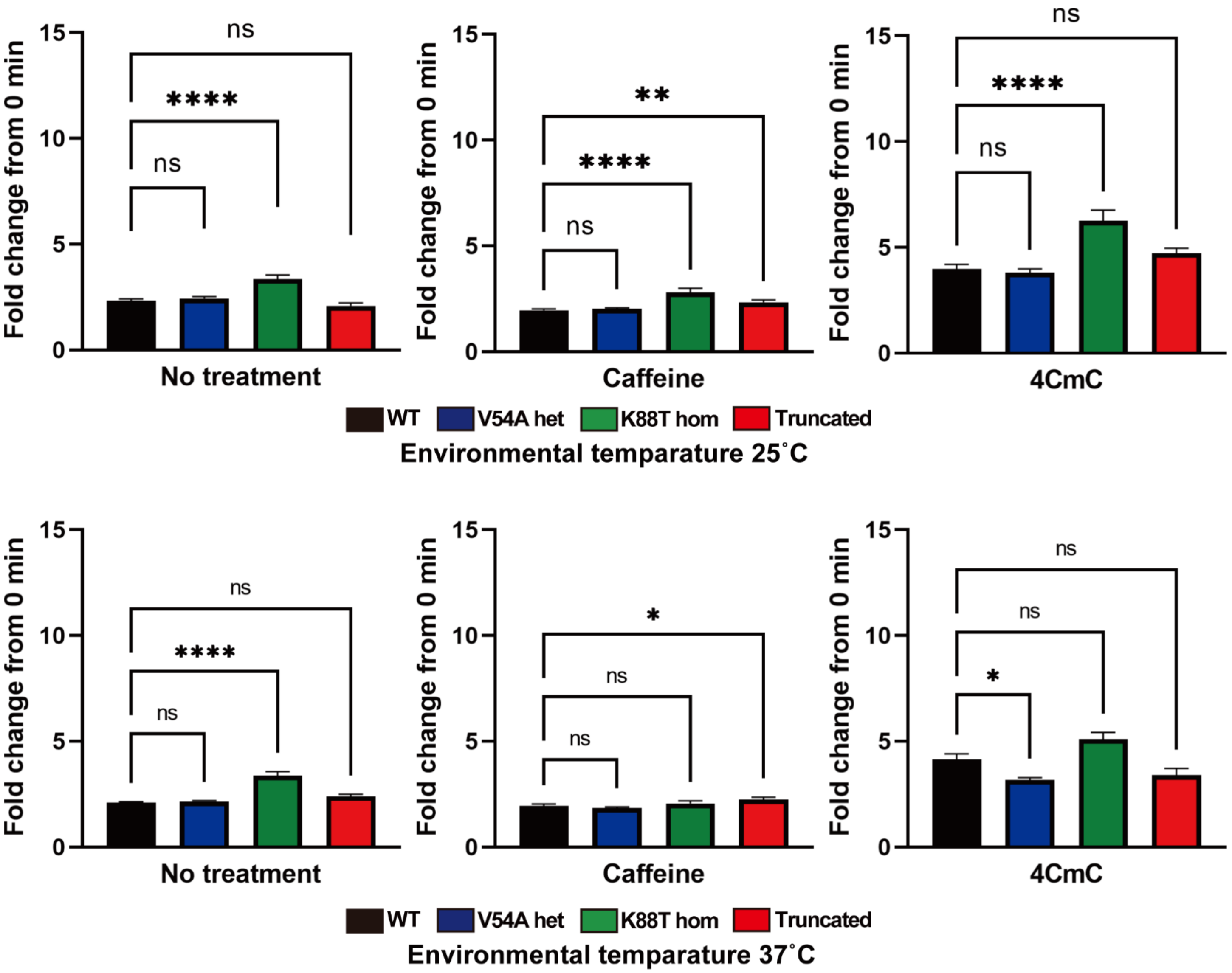

Supplementary Figure 9: ProHits-viz dotplot of AP- Dot plot of interacting partners of junctin based on AP-MS data. 20 proteins were considered high-confidence protein interactors to the bait (mCherry-junctin). Interestingly, most of the proteins listed in the top 10 are SERCA isoforms (sarco/endoplasmic reticulum Ca2+-ATPase) (*) or mitochondrial proteins (**).

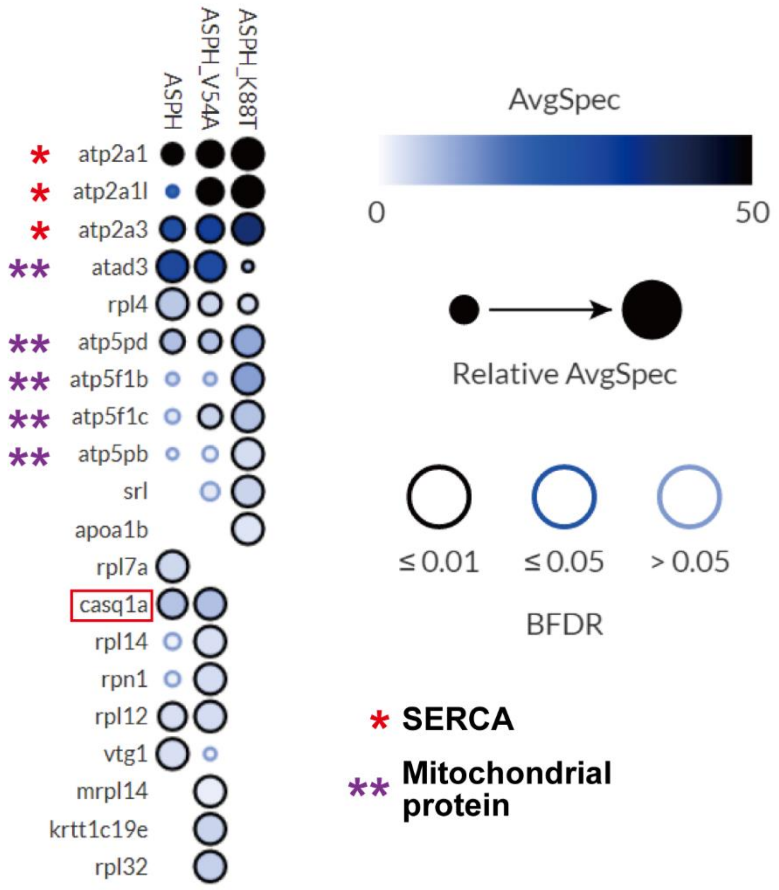

Supplementary Figure 10: SAINT Analysis outputs. SAINT analysis data of the top 10 protein interactors listed in Supplementary Figure 8. The SAINT analysis tool is used to identify high-confidence protein interactors versus control samples. Prior to applying SAINT, proteins were filtered based on iProphet >=0.95 and unique peptides >=2. iProphet cut-off is to ensure high probability of MS2 fragmentation pattern being correctly assigned to both peptide and protein. Unique peptides cut-off is to ensure we are actually seeing the protein in question. Proteins with a BFDR (Bayesian False Discovery Rate) <=0.01 are considered high-confidence protein interactors. Source data are provided as a Source Data file.

Bait	Prey	PreyGene	Spec	ctrlCounts	AvgP	MaxP	TopoAvgP	TopoMaxP	SaintScore	logOddsScore	FoldChange	BFDR
ASPH_K88T	NP_991266.1	atad3	10|5	0|0	1	1	1	1	1	8.15	75	0
ASPH	NP_991266.1	atad3	21|37	0|0	1	1	1	1	1	32.55	290	0
ASPH_V54A	NP_991266.1	atad3	32|24	0|0	1	1	1	1	1	36.95	280	0
ASPH_K88T	NP_001007030.1	atp2a1	113|133	26|24	1	1	1	1	1	55.71	4.92	0
ASPH	NP_001007030.1	atp2a1	70|102	26|24	1	1	1	1	1	6.83	3.44	0
ASPH_V54A	NP_001007030.1	atp2a1	96|113	26|24	1	1	1	1	1	37.23	4.18	0
ASPH_K88T	NP_001071001.1	atp2a1l	66|81	0|0	1	1	1	1	1	97.34	735	0
ASPH	NP_001071001.1	atp2a1l	38|0	0|0	0.5	1	0.5	1	0.5	-1.66	190	0.03
ASPH_V54A	NP_001071001.1	atp2a1l	54|65	0|0	1	1	1	1	1	80.21	595	0
ASPH_K88T	XP_697108.5	atp2a3	35|38	0|0	1	1	1	1	1	52.93	365	0
ASPH	XP_697108.5	atp2a3	22|31	0|0	1	1	1	1	1	34.02	265	0
ASPH_V54A	XP_697108.5	atp2a3	28|34	0|0	1	1	1	1	1	42.78	310	0
ASPH_K88T	NP_001019600.2	atp5f1b	10|13	0|0	1	1	1	1	1	16.08	115	0
ASPH	NP_001019600.2	atp5f1b	0|7	0|0	0.5	1	0.5	1	0.5	-1.66	35	0.15
ASPH_V54A	NP_001019600.2	atp5f1b	0|7	0|0	0.5	1	0.5	1	0.5	-1.66	35	0.15
ASPH_K88T	NP_956335.1	atp5f1c	9|5	0|0	1	1	1	1	1	8.15	70	0
ASPH	NP_956335.1	atp5f1c	0|5	0|0	0.5	1	0.5	1	0.5	-1.66	25	0.17
ASPH_V54A	NP_956335.1	atp5f1c	5|5	0|0	1	1	1	1	1	8.15	50	0
ASPH_K88T	NP_001005960.1	atp5pb	6|2	0|0	0.97	1	0.97	1	0.97	2.88	40	0
ASPH	NP_001005960.1	atp5pb	0|2	0|0	0.47	0.95	0.47	0.95	0.47	-1.66	10	0.28
ASPH_V54A	NP_001005960.1	atp5pb	0|3	0|0	0.5	0.99	0.5	0.99	0.5	-1.66	15	0.23
ASPH_K88T	NP_956996.1	atp5pd	12|9	0|0	1	1	1	1	1	14.53	105	0
ASPH	NP_956996.1	atp5pd	8|7	0|0	1	1	1	1	1	11.39	75	0
ASPH_V54A	NP_956996.1	atp5pd	4|10	0|0	1	1	1	1	1	6.47	70	0
ASPH	NP_001003620.2	casq1a	5|9	0|0	1	1	1	1	1	8.15	70	0
ASPH_V54A	NP_001003620.2	casq1a	9|6	0|0	1	1	1	1	1	9.78	75	0
ASPH_K88T	NP_998272.1	rpl4	4|3	0|0	0.99	1	0.99	1	0.99	4.72	35	0
ASPH	NP_998272.1	rpl4	5|8	0|0	1	1	1	1	1	8.15	65	0
ASPH_V54A	NP_998272.1	rpl4	3|6	0|0	1	1	1	1	1	4.72	45	0
ASPH_K88T	NP_001070999.1	srl	5|5	0|0	1	1	1	1	1	8.15	50	0
ASPH_V54A	NP_001070999.1	srl	0|5	0|0	0.5	1	0.5	1	0.5	-1.66	25	0.17
