# Supplementary material for: Variants in ASPH cause exertional heat illness and are associated with malignant hyperthermia susceptibility
Source: Nat Commun. 2022 Jun 13;13:3403. doi: 10.1038/s41467-022-31088-8 (PMC9192596; doi:10.1038/s41467-022-31088-8)
Supplement: Supplementary file 3 — Editorial Assessment Report [file 41467_2022_31088_MOESM3_ESM.pdf]

nature portfolio

GUIDED OPEN ACCESS

nature genetics | nature communications | communications biology

Contents of this report

1. Manuscript details: overview of your manuscript and the editorial team.
2. Review synthesis: summary of the reviewer reports provided by the editors.
3. Editorial recommendation: personalized evaluation and recommendation from all 3 journals.
4. Annotated reviewer comments: the referee reports with comments from the editors.
5. Open research evaluation: advice for adhering to best reproducibility practices.

About the editorial process

Because you selected the **Nature Portfolio Guided Open Access** option, your manuscript was assessed for suitability in three of our titles publishing high-quality work across the spectrum of genetics research: ***Nature Genetics***, ***Nature Communications***, and ***Communications Biology***. More information about Guided Open Access can be found here.

Collaborative editorial assessment

Your editorial team discussed the manuscript to determine its suitability for the Nature Portfolio Guided OA pilot. Our assessment of your manuscript takes into account several factors, including whether the work meets the **technical standard** of the Nature Portfolio and whether the findings are of **immediate significance** to the readership of at least one of the participating journals in the Nature Portfolio Guided Open Access genetics cluster.

Peer review

Experts were asked to evaluate the following aspects of your manuscript:

- **Novelty** in comparison to prior publications;
- **Likely audience** of researchers in terms of broad fields of study and size;
- **Potential impact** of the study on the immediate or wider research field;
- **Evidence** for the claims and whether additional experiments or analyses could feasibly strengthen the evidence;
- **Methodological detail** and whether the manuscript is reproducible as written;
- Appropriateness of the **literature review**.

Editorial evaluation of reviews

Your editorial team discussed the potential suitability of your manuscript for each of the participating journals. They then discussed the revisions necessary in order for the work to be published, keeping each journal's specific editorial criteria in mind.

Journals in the Nature portfolio will support authors wishing to transfer their reviews and (where reviewers agree) the reviewers' identities to journals outside of Springer Nature.
If you have any questions about review portability, please contact our editorial office at guidedoa@nature.com.

Manuscript details

Tracking number	Submission date	Decision date	Peer review type
GUIDEDOA-21-00332	**Nov 18, 2021**	**Jan 19, 2022**	Single-blind

Manuscript title	Author details
Variants in ASPH cause exertional heat illness and are associated with malignant hyperthermia susceptibility	James Dowling<br>**Affiliation:** Hospital for Sick Children

Editorial assessment team

**Primary editor**	**George Inglis**<br>Home journal: ***Communications Biology***<br>ORCID: **0000-0002-9069-5242**<br>Email: **george.inglis@us.nature.com**
**Other editors consulted**	**Wei Li**<br>Home journal: ***Nature Genetics***<br>ORCID: **0000-0002-7885-1775**<br><br>**Margot Brandt**<br>Home journal: ***Nature Communications***<br>ORCID: **0000-0002-9434-794X**
**About your primary editor**	George received his PhD in Genetics and Molecular Biology from Emory University, where he studied mouse models of voltage-gated sodium channel dysfunction and epilepsy. He also has research experience in epigenomics and in vitro models of neuronal development. George joined the editorial team of *Communications Biology* in September 2020 and is based in the New York office.

Editorial assessment and review synthesis

**Editor's summary and assessment**	Exertional heat illness (EHI) and malignant hyperthermia (MH) are related conditions caused by stimulus-driven skeletal muscle breakdown. Here, the authors performed whole exome or whole genome sequencing on a cohort of 103 individuals with EHI/MH and/or abnormal caffeine-halothane contracture test. They identified heterozygous pathogenic variants in the junctin isoform of the *ASPH* gene that might underlie EHI or MH susceptibility in five of these individuals, and used two orthogonal models (transgenic zebrafish and CRISPR-edited C2C12 myoblasts) to validate the pathogenicity of these variants. Altogether, they demonstrate that *ASPH* variants represent a new cause of EHI and MHS.<br><br>The editors jointly decided to send this manuscript out to review based on the integration of genetic testing and preliminary validation in multiple systems. However, the degree of advance provided and the preliminary nature of these functional experiments in zebrafish and C2C12 cells prevented further consideration by *Nature Genetics*.
**Editorial synthesis of reviewer reports**	The reviewers largely find the study to be well-designedl, but provide several comments on potential discrepancies in the datasets and figures that should be discussed in a revision. Reviewer #1 also comments on the validity of the normalization approach for swimming distance, considering some of the variability in controls on a daily basis. The reviewers also commented on the framing of the manuscript, which should be improved to promote readability and appropriate context for these results.<br><br>While *Nature Genetics* is unable to offer a revision, *Nature Communications* would be interested in considering a revision that comprehensively addresses reviewer concerns about your experimental controls and setup. *Communications Biology* would also be interested in considering a manuscript that addresses all discussion points raised by the reviewers.

Editorial recommendation

Nature Genetics<br><br>Revision not invited	The degree of advance provided or the breadth of potential interest to researchers has not matched the criteria for further consideration at *Nature Genetics*.
Nature Communications<br><br>Major revisions	A revision for *Nature Communications* would require you to fully address the concerns from Reviewer #1 regarding the variability of swimming distances in your controls; please provide additional experimental work, controls and replicates as appropriate. Your revision would also be expected to address the comments from Reviewer #3 about the promoter that you used in your assays. Please address all the other comments raised by the three Reviewers as well.
Communications Biology<br><br>Minor revisions	A revision for *Communications Biology* would only have to justify the normalization approach for swimming distances (noted by Reviewer #1) and address other discussion points raised by the reviewers. We believe these points could be addressed textually, without additional experimental evidence.

Next steps

**Editorial recommendation 1:**	Our top recommendation is to revise and resubmit your manuscript to *Nature Communications.* We feel any additional experiments necessary to respond to concerns from Reviewer #1 and #3 are reasonable to address within a 6-month time frame.
**Editorial recommendation 2:**	You may also choose to revise and resubmit your manuscript to *Communications Biology.* This option might be best if any experimental revisions are not possible/feasible at this time.
**Note**	As stated on the previous page *Nature Genetics* is not inviting a revision at this time. Please keep in mind that the journal will not be able to consider any appeals of their decision through Guided Open Access.

Revision

To follow our recommendation, please upload the revised manuscript files using **the link provided in the decision letter**.

Revision checklist

- ☐ Cover letter, stating to which journal you are submitting
- ☐ Revised manuscript
- ☐ Point-by-point response to reviews
- ☐ Updated Reporting Summary and Editorial Policy Checklist
- ☐ Supplementary materials (if applicable)

Submission elsewhere

If you choose not to follow our recommendations, you can still take the reviewer reports with you.

Option 1: Transfer to another Nature Portfolio journal
Springer Nature provides authors with the ability to transfer a manuscript within the Nature Portfolio, without the author having to upload the manuscript data again. To use this service, **please follow the transfer link provided in the decision letter.** If no link was provided, please contact guidedOA@nature.com.

> *Note that any decision to opt in to In Review at the original journal is not sent to the receiving journal on transfer. You can opt in to In Review at receiving journals that support this service by choosing to modify your manuscript on transfer.*

Option 2: Portable Peer Review option for submission to a journal outside of Nature Portfolio
If you choose to submit your revised manuscript to a journal at another publisher, we can share the reviews with another journal outside of the Nature Portfolio if requested. You will need to request that the receiving journal office contacts us at guidedOA@nature.com. We have included editorial guidance below in the reviewer reports and open research evaluation to aid in revising the manuscript for publication elsewhere.

Annotated reviewer reports

The editors have included some additional comments on specific points raised by the reviewers below, to clarify requirements for publication in the recommended journal(s). However, please note that all points should be addressed in a revision, even if an editor has not specifically commented on them.

Reviewer #1 information

**Expertise**	This reviewer has expertise in zebrafish models and muscle physiology.
**Editor's comments**	This reviewer provided a positive assessment of the manuscript, but highlighted the need to justify the normalization approach for swimming distance datasets.

Reviewer #1 comments

Section	Annotated Reviewer Comments
**Remarks to the Author: Overall significance**	This manuscript identified a candidate gene for exertional heat illness/malignant hyperthermia from two different patient cohorts. All variations in ASPH were heterozygous, suggesting a dominant effect. This and the potential impact of these variants on swimming behavior with/without heat and caffeine challenge were next investigated. The experiments using transgenic/RNA – expressing zebrafish are well done and clearly show that pathogenic variants in ASPH impact swimming behavior and muscle structure in the zebrafish model. The mechanism may be partially through excess ROS, as an antioxidant blunts the damage. Similar experiments in C2C12 cells led to similar, though less striking, results. Biochemical experiments suggest that Junctin interactions with RyR and CASQ1 may mediate Juntin's impacts on calcium homeostasis, muscle structure, and function. The impact of this manuscript is elucidating new genetic causes of EHI/MH, and identifying that the zebrafish model recapitulates important aspects of the phenotype and shows similar drug responsiveness – thus this is an excellent vertebrate model for these important conditions. Signed: Clarissa Henry
**Remarks to the Author: Strength of the claims**	The data are rigorous and well described. The authors very clearly state in which experiments they used established transgenic lines and which involved mRNA injections - showing similar results from both types of experiments bolsters their data. I only had one "major" comment - although not particularly major.<br><br>**Major:**<br>1. The variation in swimming distance is somewhat high – with controls swimming different distances (up to 25% difference) in different experiments. This is not alarming, the authors are to be lauded for showing their data clearly, but does raise the question of whether it makes sense to normalize data compared to controls on any given day in a given experiment?

	**It would be necessary to fully address this concern with further experimental work, controls and replicates as appropriate for further consideration at *Nature Communications*. Please ensure that your approach controls for heterogeneity across different days and experiments adequately.**<br><br>**It would only be necessary to either justify the current analysis or reanalyze the data to account for day-to-day variability among controls for further consideration at *Communications Biology*.**<br><br>**Minor:**<br>1. First bit of results section – relies on reader having read introduction but it's potentially worth quickly reiterating that excitation-contraction coupling (ECC) genes are strong candidates….<br>**Reviewer #2 also comments on the need for additional context, for the sake of readability.**<br><br>2. "We performed general characterization of the transgenic zebrafish lines, and identified no obvious differences in baseline motor behavior, muscle ultrastructure, or survival (Figure 2b)." Should change to Figure 2b-d<br>**Please carefully proofread the manuscript for any typos, particularly as they pertain to the figure legends. We have noted other instances in the Open Research Evaluation.**<br><br>3. Add grant number "Primary funding support was through the Canadian Institutes of Health Research (CIHR # to JJD)"<br><br>4. What does dantrolene do and how does that impact thinking about mechanisms?
**Remarks to the Author: Reproducibility**	Manuscript is well written and described.

Reviewer #2 information	
**Expertise**	This reviewer has expertise in cardiovascular and muscle physiology.
**Editor's comments**	This reviewer also provided positive feedback on the study, but introduced several important discussion points that should be addressed in a revision. They also echo some feedback from Reviewer #1 regarding the study's context, to improve readability.

Reviewer #2 comments	
**Section**	**Annotated Reviewer Comments**
**Remarks to the Author: Overall significance**	In the manuscript entitled "Variants in ASPH cause exertional heat illness and are associated with malignant hyperthermia susceptibility.", by Endo et al, the Authors described a novel function of junctin, a variant of the aspartate beta-hydroxylase (ASPH) gene, on the treatment of exertional heat illness (EHI), and malignant hyperthermia susceptibility (MHS) muscle diseases. In the well-written and clear manuscript, the Authors showed that specific mutations found in human muscle samples on junctin (K88T and V54A) are relevant to keep the stability of ryanodine receptors during the excitation-contraction coupling, especially during high temperature and under the effect of halothane. The interesting approach taken by the Authors to understand these mutations was to transfer the mutated gene into two different and wide-used pre-clinical muscle models: zebrafish and C2C12 cell line. The findings in this manuscript are clinically relevant for the EHI and MHS patients, and for finding new alternative approaches for those diseases. There are nevertheless, minor comments that the Authors should address before a final decision.<br><br>1) The Authors should give the (in extenso) name of the gene abbreviation.<br><br>2) At the end of the introduction, the Authors only mentioned junction as one candidate for EHI/ MHS, without describing how this gene was selected, the function of the protein, and to whom it interacts. These pieces of information are necessary to understand the reason for the work, especially for those readers who are not familiar with the topic. Part of the explanation was found in the results, but still should be present in the introduction for better comprehension of the manuscript.<br>**This point (and point #3) echoes similar feedback from Reviewer #1 regarding the context of the manuscript (Minor Point #1).**<br><br>3) From the introduction, it is difficult to clearly see the aim of the work. The Authors should elaborate a sentence defining the aim(s) and the pieces of evidence to support the aim(s).

	4) The Authors should describe in the results the concentration of the drugs used in the study. Moreover, it is lacking on figures 2 and 3 a description of the statistical analyses of whether the pharmacological treatment did or did not produce a significant result different from the control condition.<br>**For the sake of reproducibility, please carefully elaborate on the statistical analyses for these results.**<br><br>5) In-text for figure 2, the Authors state ", and identified no obvious differences in baseline motor behavior, muscle ultrastructure, or survival (Figure 2B)." Although there are no differences, the Authors only showed muscle ultrastructure data, without the other parameters. Maybe just a re-adjust of the position of the label (Figure 2B) will correct the imprecision of the text.<br>**It would be necessary to elaborate on this point for future consideration at *Nature Communications* and *Communications Biology.***<br><br>6) The Authors should clarify in the results whether the experiments using C2C12 cells were done in myoblast or myotubes, and if so, the passage, confluency, and days after differentiation.<br>**The Methods should include sufficient detail for others to repeat these experiments. Please note that we do not have a word limit for the Methods section.**<br><br>7) In Figure 4e, the truncated data is represented with a red bar, instead of the orange bar from the other experiments. Is there a specific reason for the change of color?<br><br>8) In the paragraph of the discussion, starting with "Our data expand the knowledge… and positive CHCT in his sibling" the Authors should provide the missing citations.
**Remarks to the Author: Impact**	The current and the following works on the function of junctin will provide important and relevant pre-clinical and clinical information on the new therapies for EHI and MHS.
**Remarks to the Author: Strength of the claims**	The data presented in the current manuscript are enough to convince for further studies of junctin related to EHI and MHS.
**Remarks to the Author: Reproducibility**	The Authors took care of the statistical analyses, giving enough information for the continuity work from independent groups.

Reviewer #3 information

**Expertise**	This reviewer has expertise in zebrafish models of cardiovascular disease.
**Editor's comments**	While we recognize this review is brief, the reviewer noted some important discrepancies in the manuscript that should be discussed in a revision.

Reviewer #3 comments

**Section**	**Annotated Reviewer Comments**
**Remarks to the Author: Overall significance**	In this manuscript, the authors integrated human genetics, zebrafish genetics and cell culture models to discover causative genes for exertional heat illness (EHI) and malignant hyperthermia (MH). They performed genomic sequencing on a cohort with EHI/MH and identified rare, pathogenic variants in ASPH. They then generated transgenic zebrafish for two of the variants, which recapitulated the corresponding phenotypes in human. They went on to generate knock-in alleles in C2C12 cell and also obtained promising data to prove causality. The logical flow is clear, and data and their conclusions are convincing, and the presentation is excellent.
**Remarks to the Author: Impact**	Successful modeling of the disease in the efficient zebrafish model is interesting, which shall have great potential for testing additional variants that will be identified in the future. Thus, the impact could be high.
**Remarks to the Author: Strength of the claims**	I do have the following concerns.<br>1. As to the transgenic fish, please comment on the promoter that you used. Whether the promoter drives gene expression in somites or ubiquitously in the whole body?<br>**Addressing this point with adequate experimental evidence would be necessary for further consideration at *Nature Communications*, though it would be sufficient to cite relevant literature for revision at *Communications Biology*.**<br><br>2. Page 6, the end of the second paragraph: WT and K88T junction mRNA levels were comparable, V54A mRNA was reduced. Please provide some explanation on this phenomenon?<br>**Discussion of this point would be necessary for further consideration at *Nature Communications* and *Communications Biology*.**<br><br>3. Fig. 3A and 4B. Are the units the same? If so, please choose one. It seems data in fish and cell are different. Why?<br>**Please clarify the units between these figures, for the sake of consistency.**

**Remarks to the Author: Reproducibility**	Statistical analysis is adequate.

Open research evaluation

General information

Guidelines for Transparency and Openness Promotion (TOP) in Journal Policies and Practices ("TOP Guidelines")

The recommendations and requests in the table below are aimed at bringing your manuscript in line with common community standards as exemplified by the TOP Guidelines. While every publisher and journal will implement these guidelines differently, the recommendations below are all consistent with the policies at Nature Portfolio. In most cases, these will align with TOP Guidelines Level 2.

FAIR Principles

The goal of the recommendations in the table below related to **data or code** availability is to promote the FAIR Guiding Principles for scientific data management and stewardship (*Scientific Data* **3**: 160018, 2016). The FAIR Principles are a set of guidelines for improving 4 important aspects of digital research objects: **F**indability, **A**ccessibility, **I**nteroperability and **R**eusability.

ORCID

ORCID is a non-profit organization that provides researchers with a unique digital identifier. These identifiers can be used by editors, funding agencies, publishers, and institutions to reliably identify individuals in the same way that ISBNs and DOIs identify books and articles. Thus the risk of confusing your identity with another researcher with the same name is eliminated. **The ORCID website** provides researchers with a page where your comprehensive research activity can be stored.

Springer Nature collaborates with the ORCID organization to ensure that your research contributions (as authors and peer reviewers) are correctly attributed to you. Learn more at https://www.springernature.com/gp/researchers/orcid

Data availability

Data Availability Statement

Many journals, including all Nature Portfolio journals, require a Data Availability Statement in the manuscript as a condition of publication. The Data Availability Statement should be as detailed as possible and include accession codes or other unique IDs for deposited data, information about where source data can be found, and specify any restrictions to data access that may apply. At a minimum, the statement should indicate that data are available upon request and explain how data access can be granted. If data access is not possible, the reasons for this must be made clear in the Data Availability Statement.

More information about the Nature Portfolio data availability policy can be found here: https://www.nature.com/nature-portfolio/editorial-policies/reporting-standards#availability-of-data

Additional information about Data Availability Statements and Springer Nature's data policies are available here: http://www.springernature.com/gp/authors/research-data-policy/data-availability-statements/12330880

Mandatory data deposition

Most scientific journals, including all Nature Portfolio journals, require that any newly-generated DNA sequence data must be made publicly available before publication. There are some exceptions allowed for sensitive clinical data, but this should be discussed with the editor. All data must be deposited in a community-approved repository and accession codes/unique IDs must be included within the Data Availability Statement in the manuscript.

Examples of appropriate public repositories are listed below:

- GenBank
- Sequence Read Archive (WGS or WES data)
- The European Nucleotide Archive (ENA)

More information on mandatory data deposition policies at the Nature Portfolio can be found at http://www.nature.com/authors/policies/availability.html#data

Please visit https://www.springernature.com/gp/authors/research-data-policy/repositories/12327124 for a list of approved repositories for various data types.

Other data requests

We strongly encourage the deposition of your full microscopy image data sets in the Image Data Resource: https://idr.openmicroscopy.org/about

All source data underlying the graphs and charts presented in the main figures must be made available as Supplementary Data (in Excel or text format) or via a generalist repository (eg, Figshare or Dryad). This is mandatory for publication in a Nature Portfolio journal, but is also best practice for publication in any venue.

The following figures require associated source data: Fig 2d-e, 3a-c, 3e, 4b-e

Ethics

We believe that authors, peer reviewers and editors should be required to disclose any competing interests that might influence their decisions and conclusions around a particular piece of content. In the interests of transparency and to help readers form their own judgements of potential bias, Nature Portfolio journals require authors to declare any competing financial and/or non-financial interests in relation to the work described.

Please provide a 'Competing interests' statement using one of the following standard sentences:

1. The authors declare the following competing interests: [specify competing interests]
2. The authors declare no competing interests.

See the Nature Portfolio competing interests policy for further information:
https://www.nature.com/nature-research/editorial-policies/competing-interests

The Springer Nature policy can be found here:
https://www.springernature.com/gp/policies/editorial-policies

We believe that research that involves the use of clinical, biomedical or biometric data from human participants must only be carried out with the explicit consent of those whose data are involved. Consent must be obtained without any form of coercion and with participants' explicit understanding of the purpose for which their data will be used.

Because your study includes human participants, confirmation that all relevant ethical regulations were followed is needed for publication in any Springer Nature journal, and that **informed consent** was obtained. This must be stated in the Methods section, including the name of the board and institution that approved the study protocol.

Further details about the Nature Portfolio policy can be found at
https://www.nature.com/commsbio/editorial-policies/ethics-and-biosecurity

We believe that Springer Nature has a responsibility to support the relevant guidelines (based on research community or geographical region) that specify best practice in research and thus require all experimental results on animal and human participants to conform to the authors' local regulations and ethical standards, and we also encourage adherence to international standards.

Because your study uses live vertebrates, a statement affirming that you have complied with all relevant ethical regulations for animal testing and research is necessary. A statement explicitly confirming if the study received ethical approval, including the name of the board and institution that approved the study protocol is also required. The species, strain, sex and age of animals should be included.

Further details on our policies can be found at
https://www.nature.com/commsbio/editorial-policies/ethics-and-biosecurity

Reporting & reproducibility

All life science papers published in Nature Portfolio journals require submission of unprocessed original images of gels and western blots to be submitted with the final accepted version in order to promote data transparency. These unprocessed images are published in the Supplementary Information.

Please include the full, uncropped blot/gel images for **Fig 4a and Supp Fig 5a** as new Supplementary Figures, which should be cited in the main manuscript text.

For more information about our image integrity policies, see
https://www.nature.com/commsbio/editorial-policies/image-integrity#electrophoretic-gels-and-blots

Cell line misidentification and cross-contamination is a common problem with serious consequences. Authors are asked to report on the source and authentication of their cell lines.

Materials availability

We recommend that you deposit your newly generated plasmids in a community repository, such as https://www.addgene.org/, to support open research efforts.

Nature Portfolio supports the Resource Identification Initiative (https://www.force11.org/group/resource-identification-initiative), with the aim of promoting unique, persistent identification and tracking of key biological resources, including antibodies, cell lines, model organisms and tools.

We encourage authors to include unique identifiers provided by the Resource Identification Portal, (RRIDs; for example, Antibody: RRID:AB_2140114; Organism: RRID:MGI_MGI:3840442), in the manuscript. More information on how to include listed RRIDs or generate new RRIDs can be found on the Resource Identification Portal:
https://scicrunch.org/resources/about/Getting%20Started

We strongly encourage deposition of new cell lines in repositories that will distribute them with certificates of authentication. Alternatively, we recommend that authors establish a profile of their new cell lines to allow future authentication. The distribution of human cell lines used in research should not be hindered by restrictions from donors. Researchers developing cell lines must investigate and disclose any restrictions associated with the tissue they are using.

Statistical reporting

Wherever statistics have been derived (e.g. error bars, box plots, statistical significance) figure legends should provide and define the n number (i.e. the sample size used to derive statistics) as a precise value (not a range), using the wording "n=X biologically independent samples/animals/cells/independent experiments/n= X cells examined over Y independent experiments" etc. as applicable. The figure legends must also indicate the statistical test used. Where appropriate, please indicate in the figure legends whether the statistical tests were one-sided or two-sided and whether adjustments were made for multiple comparisons. For null hypothesis testing, please indicate the test statistic (e.g. F, t, r) with confidence intervals, effect sizes, degrees of freedom and P values noted. **Please update Fig 3e and 4e accordingly.**

All error bars need to be defined in the figure legends (e.g. SD, SEM) together with a measure of centre (e.g. mean, median). For example, the legends should state something along the lines of "Data are presented as mean values +/- SEM" as appropriate. All box plots need to be defined in the legends in terms of minima, maxima, centre, bounds of box and whiskers and percentile. **Please update Supp Fig 5b accordingly.**

For examples of expected description of statistics in figure legends, please see the following: https://www.nature.com/articles/s41467-019-11636-5 or https://www.nature.com/articles/s41467-019-11510-4.

When describing results as "significant" in the main text, please include details about the statistical test used and provide an exact p-value, rather than a significance threshold. **Please define what */**/**** means in each figure legend, and provide exact *p*-values when possible, in Fig 2d-e, 3b-c, 3e, 4c-e, and Supp Fig 6.**

Please note that statistics such as error bars significance and p values cannot be derived from n<3 and must be removed in all such cases.

We strongly discourage deriving statistics from technical replicates, unless there is a clear scientific justification for why providing this information is important. Conflating technical and biological variability, e.g., by pooling technically replicates samples across independent experiments is strongly discouraged.

For examples of expected description of statistics in figure legends, please see the following: https://www.nature.com/articles/s41467-019-11636-5 or https://www.nature.com/articles/s41467-019-11510-4.

Data presentation
Bar graphs should only be used to present counts or proportions. If you are using bar graphs that present means/averages, it is best practice to include individual data points and/or convert the graph to a boxplot or dot-plot. You may wish to refer to this blog post (https://ecrlife420999811.wordpress.com/2018/07/10/beyond-bar-graphs-free-tools-and-resources-for-creating-more-transparent-figures-for-small-datasets/) about representing data distribution in plots (particularly for small datasets).<br><br>**Please update Fig 3b, 4c-e, and Supp Fig 3, 6 accordingly.**
Please ensure that all microscopy images and photographs include a scale bar and this scale bar is defined on the panels or in the figure legends.
Please state in the figure legends how many times each experiment was repeated independently with similar results. This is needed for all experiments, but is particularly important wherever results from representative experiments (such as micrographs) are shown. If space in the legends is limiting, this information can be included in a section titled “Statistics and Reproducibility” in the methods section.
All blots/gels must be accompanied by size markers in every figure panel. In addition, please check that your blot/gel images comply with the Nature Portfolio image integrity guidelines: https://www.nature.com/nature-research/editorial-policies/image-integrity<br><br>**Please update Fig 4a accordingly.**
**Please note the following errors in figure legends:**<br>● The legend for Table 1 is missing.<br>● The legends for Figures 1a and 1b are incorrectly labelled as “1b, c”.<br>● Supplementary Table 3 is referenced in the legend of Fig 3a, instead of Supplementary Table 4. Please rectify this in the legend of Figure 3a.
